# Efficiency and performance tests of the sorptive building materials that reduce indoor formaldehyde concentrations

**DOI:** 10.1371/journal.pone.0210416

**Published:** 2019-01-24

**Authors:** Kun-Chih Huang, Yaw-Shyan Tsay, Fang-Ming Lin, Ching-Chang Lee, Jung-Wei Chang

**Affiliations:** 1 Department of Architecture, College of Engineering, National Cheng Kung University, Tainan, Taiwan; 2 Department of Wood Science and Design, National Pingtung University of Science and Technology, Pingtung, Taiwan; 3 Department of Environmental and Occupational Health, Medical College, National Cheng Kung University, Tainan, Taiwan; 4 Institute of Environmental and Occupational Health Sciences, School of Medicine, National Yang-Ming University, Taipei, Taiwan; National Sun Yat-sen University, TAIWAN

## Abstract

The adsorption of volatile organic compounds by building materials reduces the pollutant concentrations in indoor air. We collected three interior building materials with adsorption potentials—latex paint, micro-carbonized plywood, and moisture-buffering siding—used the sorptive building materials test (SBMT) to determine how much they reduced indoor formaldehyde (HCHO) concentrations, and then assessed the consequent reduction in human cancer risk from HCHO inhalation. Adsorption of HCHO by building materials significantly improved the effective ventilation efficiency. For example, the equivalent ventilation rate for Celite siding—used for humidity control—was 1.44 m^3^/(m^2^·h) at 25°C, 50% relative humidity (RH); the loading factor (L) was 0.4 m^2^/m^3^, and the HCHO concentration was 0.2 ppm; this effect is equivalent to a higher ventilation rate of approximately 0.6 air changes per hour in a typical Taiwanese dwelling. There was also a substantial reduction of risk in Case MCP-2 (C_in,te_: 245 μg/m^3^, 30°C, 50% RH): males: down 5.73 × 10^−4^; females: down 4.84 × 10^−4^). The selection of adsorptive building materials for interior surfaces, therefore, significantly reduces human inhalation of HCHO. Our findings should encourage developing and using innovative building materials that help improve indoor air quality and thus provide building occupants with healthier working and living environments.

## Introduction

Current studies hypothesize that many building materials affect human health, including those that adsorb and reduce the level of indoor chemical pollutants. Many interior decoration materials, furniture, and electronic equipment contain toxic substances such as formaldehyde (HCHO) and toluene, which are easily emitted and seriously harm the health of people exposed to them for a long time in indoor environments [1–3].

Well-controlled indoor air quality (IAQ) and indoor environmental health (IEH) reduces the health risks posed by the toxic gases emitted by building materials. The surface characteristics of microporous building materials can change in response to ambient temperature and humidity and affect their adsorption and desorption behaviors. Several studies have found that the adsorption of air pollutants by building materials reduces the indoor concentration and changes the distribution characteristics of volatile organic compounds (VOCs); in general, the adsorption of VOCs by building materials falls at higher temperatures [4,5]. Water vapor prevents temperature from increasing with VOCs adsorption equilibria; when the relative humidity (RH) increases, the adsorption of VOCs by building materials decreases, but it increases with polarity increases in VOC molecules [6,7].

The adsorption and desorption behaviors of building materials might transform the removal of indoor secondary pollutants, and for several months or even years turn into a chronic hazard for occupants [8, 9]. Using the building materials as the adsorbents, the most important characteristics are adsorption specific surface area, pore-size distribution, functional groups, and polarity. The adsorption/desorption performance of the building materials is illustrated in S1 Fig. VOC emissions depend not only on the physical properties of the building materials-pollutant interaction, but also on environmental conditions, especially temperature and humidity. Several studies have actively discussed the effects of temperature and humidity on the partition and diffusion of HCHO in sorption materials [10–14]. Taiwan is in a subtropical region with high temperatures and RH. Therefore, it is necessary to evaluate the association between the environment and the adsorption of building materials.

Since 2004, the Taiwan Architecture and Building Research Institute has promoted the Green Building Materials label (GBML) initiative to establish domestic policies for improving control of GBMs. Considering the effects of IAQ on human health, Taiwan’s Building Technical Regulations requires the percentage of GBMs to increase from 30% to 45% for indoor decoration. Until now, healthy GBMs (focusing on low indoor pollution) provide substantial passive protection despite a large amount of VOCs being released from many indoor decorations made of GBMs. Therefore, using innovative sorptive building materials (SBMs) might help reduce not only the amount of energy consumption required to operate ventilation equipment but also the concentrations of indoor pollutants. This approach needs no special equipment. Overall, it can increase the efficiency of purging indoor air pollutants like HCHO, and then create healthier working and living environments.

Methods for controlling and removing indoor air pollutants are becoming more important [15–16]. A review of expert assessments provides a coherent interpretation of available data about the health hazards of HCHO [17–25]. Inhaling HCHO causes ocular, nasal, and respiratory irritations, as well as nasopharyngeal carcinoma in humans, and its carcinogenicity is linked to irritant effects [26]. The mode of action suggests a threshold for nasopharyngeal carcinogenic effects, based on a dual mode of action (cytotoxicity and genotoxicity). At the lowest concentrations, the critical effects of HCHO in humans are irritations of the eyes and respiratory tract.

Epidemiological studies have also found a link between long-term human exposure to high levels of HCHO and cancers in the nose, mouth, throat, digestive system, lungs, skin, and blood [27]. Therefore, HCHO is a high-risk air pollutant for humans, and the increase in inhalation cancer risks is a significant public health problem.

To facilitate the quality control inspection for wood-based materials, governments and international organizations use several standard to evaluate HCHO emissions. Some well-known environmental chamber methods include ASTM D 6007–2, ASTM E 1333, EN 717–1, ISO 16000–23, and JIS 1905–1[28–32], most of which are used and promoted by developed countries [33–35]. The Taiwan government formally announced its own new indoor air standard, CNS 16000–23, in December 2011 (S1 Table).

We used the sorptive building materials test (SBMT) method to reduce HCHO concentrations, and to understand how different building materials and environmental conditions affect the risk of inhalation cancer from exposure to HCHO.

## Materials and methods

### Evaluating airborne formaldehyde reduction

Performance tests of the efficacy of SBMs for reducing airborne HCHO in air were done using a 99-liter stainless steel chamber (S2 Table). The formaldehyde-spiked air was used to determine the performance of different boards and architectural paint in reducing formaldehyde concentrations. The materials we tested included latex paint (LP), new technology, developments for micro-carbonized plywood (MCP), and Japan’s humidity-controlling material for Celite siding (CS) based on the ISO 16000 reference standard (the same as Taiwan’s Chinese National Standards [CNS]) (S3 Table). Specific suppliers in Taiwan developed the three materials selected in this study to purify air; therefore, high-temperature MCP and porous nano-molecules were added to them. However, there are still not enough data to create a validation method for these building materials in Taiwan, but more products that claim similar adsorption functions will enter the market. We first selected three common materials to verify the effectiveness of our method, and we hope that others will conduct more research to establish the needed standards.

With this structural design, HCHO molecules attach to the porous structure of the building material, and then accumulate and are easily adsorbed. The test samples were 0.2 m × 0.2 m squares; their edges were sealed with aluminum foil. The product loading factor [*L*] was 0.4 m^2^/m^3^. The sample was maintained in a 4°C environment to preclude pollution. We followed the quality assurance and quality control (QA/QC) parameters outlined in ISO 16000–11:2006 when transporting and storing samples, and when performing the experiments [36].

The capabilities of sorptive building materials in different environmental situations were evaluated using Taiwan’s national CNS 16000–23 (25°C, 50% RH) and at 30°C and 75% RH. The levels of formaldehyde to be injected into the stainless steel chamber were set at 0.1 and 0.2 ppm, based on Taiwan Indoor Air Quality Management Act in 2011(S3 Table).

### Experimental conditions

To control VOC concentrations, small-scale chamber equipment and the sorptive building material test system (SBMTS) at the National Performance Laboratory Center in Taiwan [16, 37] were prepared based on ISO 16000-23/CNS 16000–23 standards. We also evaluated the efficacy of the airborne HCHO reduction of building materials under different temperatures and relative humidities. Before a test, the empty chamber was vented for 12 h, and then the background HCHO concentration was measured. The background concentration had to be low enough not to interfere with the test. Then the test chamber was flushed with HCHO-spiked air. At least five air exchanges were required before the test specimen was placed in the chamber. We used an air cylinder to ensure that the contaminant levels in the chamber air were no higher than required. The exhaust from the test chamber was sucked into a fume hood to avoid contaminating the laboratory. After each test, we washed the inner surface of the test chamber with a detergent and then rinsed it twice with freshly distilled water. The chamber was then dried and purged under test conditions. We kept the concentration of the background VOCs below 10 μg/m^3^ and of the background HCHO below 2 μg/m^3^. In addition, we ensured that the purified water used for humidification did not interfere with the performance tests.

Unwanted moisture and hazardous substances were eliminated using moisture traps and carbon filters, and clean, dry air was provided by stably flowing gas generators. The HCHO ventilation rate was 0.5 h^‒1^, which ensured a stable concentration of 0.1 to 0.2 ppm inside the chamber (S2 Fig).

Pumps fitted with a DNPH (2,4-dinitrophenylhydrazine) cartridge drew air samples from the inlet and outlet ports of the test chamber at predefined elapsed times (*t*_e_) at a rate of 100 mL/min for 2 h. The samples were stored in a refrigerator at 4°C for 8 h, then analyzed using high performance liquid chromatography (HPLC). Air samples were collected every 2 h for the first 12 h, and then at 24 ± 2 h and 72 ± 6 h. During the sampling, we checked the total airflow through the test chamber to ensure that there were no air leaks. The outlet airflow rate during air sampling was equal to the inlet airflow rate minus the sum of the sampling airflow rates. The remaining outlet airflow rate was no less than 20% of the total sampling airflow rate. In addition to the short-term tests, we conducted long-term tests to allow reductions to equal one-half of the initial values. The results could be used to predict the lifetime performance of SBMs.

The subsequent reemission of HCHO from the materials was evaluated by purging the chamber with clean air after 96 h. The input and output HCHO concentrations were checked during reemission. We measured the quantity of HCHO removed from the test chamber air for each test specimen (S3 Table).

### Evaluating the sorption flux of sorptive building materials

The HCHO reduction of SBMs was evaluated using sorption flux, an equivalent ventilation rate, and the adsorption rate provided in international standards (ISO 16000–11:2006). In addition, the HCHO concentration differences between the inlet and outlet of the test chamber was evaluated. The important assumption of all the following equations is “steady-state”, which allows the adsorption rate of a SBM to be calculated from the difference between inlet and outlet HCHO concentrations. HCHO transport into the chamber is less likely to be controlled by molecular diffusion because the inflow is preset. If the adsorption rate is constant, the HCHO concentration in the chamber will also be constant, because HCHO-spiked air is continuously supplied [38, 39]. In this study, based on the sampling stage, 12 h was needed to reach the steady state before sampling. We sampled the air every 2 h for the first 24 h and then collected samples every 24 h.

#### Calculations of sorption flux, SB_m_, and SB_v,eq_ were based on ISO and JIS standards

The sorption flux *SB*_m_ (μg/m^2^·h) was calculated from the concentration difference between the inlet and the outlet of the test chamber, the airflow rate *Q*_*v*_ (m^3^/h) of the test chamber, and the surface area (A) of the SBM (Eq 1):

$$SB_{m}=\frac{(C_{in,t_{e}}-C_{out,t_{e}})Q_{v}}{A}$$ Eq (1)

where *t*_e_ is the time from the start of the test to the start of air sampling, *C*_*in*_ (μg/m^3^) is the HCHO concentration at the test chamber inlet at *t*_e_, *C*_*out*_ (μg/m^3^) is the HCHO concentration at the test chamber outlet at *t*_e_, *Q*_*v*_ (m^3^/h) is the air flow rate of the test chamber, and *A* (m^2^) is the surface area of the tested SBM.

$$SB_{v,eq}=\frac{\left(\frac{C_{in,t_{e}}}{C_{out,t_{e}}}-1\right)Q_{v}}{A}$$ Eq (2)

To describe the reduction of pollution in the room, the sorption flux (*SB*_m_) (μg/m^2^·h) was converted to the equivalent ventilation rate (*SB*_*v*,*eq*_) (m^3^/m^2^·h), which allows a comparison between the effect of ventilation and passive adsorption. To arrive at the same HCHO reduction rate, the clean air ventilation rate must increase based on the SBM used. The adsorption rate (SB_*r*_) (%) was evaluated based on the inlet and outlet HCHO concentration difference, calculated using Eq (3). Additionally, we used Eq (4) to calculate the equivalent clean air ventilation rate:

$$SB_{r}=\frac{\left(C_{in,t_{e}}-C_{out,t_{e}}\right)}{C_{in,t_{e}}}\times 100\%$$ Eq (3)

$$\mathrm{ACH}=\frac{\left({\mathrm{SB}}_{v,\mathrm{eq}}\right)A}{V}=\left({\mathrm{SB}}_{v,\mathrm{eq}}\right)\times L$$ Eq (4)

where ACH is air changes per hour; *SB*_*r*_ (%) is the average adsorption rate of total flux at *t*_e_; *V* (m^3^) is the volume of the test chamber; and *L* (m^2^/m^3^) is the product loading factor in the space. The loading factor determines how large the test specimens are relative to the volume of the test chamber or the room. A higher loading factor means a larger emissions source and thus higher emissions.

When a building material is used as the adsorbent, its most important characteristic is its intrinsic adsorption ability, not its specific surface area, pore-size distribution, functional groups, or polarity. Moreover, HCHO adsorption was analyzed using a continuous photoacoustic multi-gas monitor (Innvoa 1312; Bruel & Kjaer, Naerum, Denmark). We also did recovery tests in the empty chamber and compared outlet and inlet ports. All mean recoveries were greater than 95%.

The precision and accuracy of the calibration curve met ISO 16000–3 requirements: the calibration of low-to-high concentrations of HCHO ranged from 0.048 to 15.768 μg, and R^2^ was more than 0.995. The mean low- and high-concentration calibration recoveries were, respectively, 100.64% and 99.36%. Precision and accuracy were ± 15% and the relative standard deviation was < 10%. To ensure that the test conditions were stable throughout the long-term tests, blanks and recovery samples were checked before the test. Before testing the semi-automatic air supply equipment, 5 times the needed volume of fresh air for one day was provided to clean up the inside. The background concentrations equaled standard values by ISO 16000–23:2009, because concentrations of HCHO were lower than 2 μg/m^3^ and of TVOCs were lower than 20 μg/m^3^. Aluminum foil was pasted along the edge of the sample area. To meet the laboratory QA and QC standards of the transportation, storage, and other procedures, the samples were kept in a 25°C-environment to prevent pollution. To evaluate the adsorption performance of building materials, the average of formaldehyde concentration will be calculated from 3 times sampling for each test. Moreover, under different condition, the formaldehyde concentration of each test will also be carried out 3 times. In order to ensure the formaldehyde concentration of the inlet and outlet in each test, we also monitor the concentration through the monitoring instrument to confirm the accuracy for the formaldehyde concentration can be reached.

In the preliminary test, we first verified the efficacy of the testing method. Long-term tests might take more than one month. Moreover, the total amount of HCHO adsorption by SBMs can be obtained using a small-scale environmental chamber, so most results came from the chamber. If testing took more than one week, we will also do a long-term test. We developed a modified sorption breakthrough capacity method system [40]. After a column was filled with ground adsorbent, the amount of adsorption was measured. The HCHO sorption isotherm was established using the breakthrough test, and we were able to estimate the sorption capacity of building materials using a regression curve.

#### Tube-test for long-term HCHO reduction

We ground the test specimen into fragments that maintained their secondary structure and fit in the sample tube. Fine particles were removed before the test. The test specimen was vacuum dried because absolute dryness is fundamental. Before the sample tube was connected to the system, the HCHO concentration of the spiked air was determined. The HCHO air flow was begun and continued until the outlet air contained a 0.5% concentration of HCHO-spiked air. The sorption capacity (w_s_) (saturated HCHO mass**/**mass of sorbent) at that concentration. This is considered the breakthrough time (*t*_*b*_).

We first calculated the HCHO w_s_ of the test specimen using Eq (5):

$$w_{s}=\frac{\rho_{s}\times q_{s}\times t_{b}}{1000\times m}$$ Eq (5)

where *ρ*_s_ is the HCHO concentration in the HCHO-spiked air; *q*_s_ is the air flow rate; *t*_*b*_ is the breakthrough time; and *m* is the mass of the test specimen in the sample tube.

Then we calculated the saturation mass**/**area (*ρ*_*Aa*_) using Eq (6):

$$\rho_{Aa}=w_{s}\times \rho_{a}$$ Eq (6)

where *ρ*_*A*_ is the surface density of the sorptive material calculated as its mass**/**surface area.

To test for long-term HCHO reduction, we determined the point at which the SBM was most efficient at reducing HCHO concentrations and then measure the total mass**/**area of sorption (*ρ*_*Ac*_) of HCHO and the elapsed time (*t*_*e*_).

We used Eq (7) to calculate the equivalent ventilation rate per area:

$$SB_{m}=\frac{(C_{in,t_{e}}-C_{out,t_{e}})Q_{v}}{A}$$ Eq (7)

We can calculate the total mass**/**area of sorption (*ρ*_*Ac*_) at the half-lifetime:

$$\rho_{Ac}=\sum_{i}(SB_{m,i}\times \Delta t_{e,i})$$ Eq (8)

where Δ*t*_e,i_ = *t*_*e*,*i*_ ‒ *t*_*e*,*i*‒1_.

The test method is used to estimate the *w*_*s*_ of two types of SBMs when the HCHO concentration is 0.1 ppm. The surface density of the material is converted into the total mass**/**surface area. The total amount of the sample tube is estimated to be converted into a small-sized chamber. The total number of days of formaldehyde sorption can be estimated to obtain the long-term effective duration of the SBMs (S6 Table). The conversion method is the following two methods:

$$\frac{\rho_{Aa}}{\rho_{Ac}}\times \Delta t_{e,i}=t_{lt}\left(day\right)$$ Eq (9)

*t*_*lt*_: lifetime of the pollutant-removing performance

### Health risk assessment

Cancer risks posed by indoor personal exposure to formaldehyde can be calculated by using Eqs (10–12) [29]:

$$C_{steady}=\frac{\mathrm{ER}\times \mathrm{Area}}{\mathrm{ACH}\times \mathrm{VOL}\times K}$$ (10)

$$Risk=\frac{C_{steady}\times IR\times AF\times ED}{BW\times AT}\left(mg/kg/day\right)\times Potency\;Factors{\left(mg/kg/day\right)}^{-1}$$ (11)

$$Risk_{reduction}=\frac{(C_{in,te-}C_{steady})\times IR\times AF\times ED}{BW\times AT}\times Potency\;Factors{\left(mg/kg/day\right)}^{-1}$$ (12)

Incomplete mixing is often adjusted for by introducing an empirical mixing factor (K), which depends upon the relative locations of sources and receptors, the shape and size of the indoor space, and ventilation parameters. In this study, K is assumed to be 0.5 [41].

The concentrations of HCHO in a representative Taiwan dwelling model room were calculated based on Eq (10) [42], where ER is the emission rate from a surface (mg/m^2^/h) calculated from HCHO concentrations measured in the chamber; Area is the area of a surface (wall, floor, ceiling) (m^2^); VOL is the volume of the room (m^3^); ACH is the air-change rate (h^−1^); and K is the mixing factor. This formula is based on several assumptions, including steady-state emission from the products, which require constant air temperature and humidity. Moreover, houses are assumed to be single well-mixed zones. Outdoor concentrations of HCHO were omitted. We calculated risks by multiplying chronic daily intake and Inhalation Cancer Potency Factors. The Inhalation Cancer Potency Factors for HCHO in this system is 2.0 × 10^−2^ (mg/kg/day)^‒1^ [43]. In addition, the absorption rate is assumed to be 100% [44]. Distributions of the time that adult males and females spend in dwelling were taken from Time Use Patterns in Taiwan [45], where people on average spend 14 h per day in their dwelling. Other exposure factors, such as the inhalation rate (m^3^/h), exposure time (hours/days), exposure frequency (days/years), exposure duration (years), body weight (kg), and the average lifetime (years), were derived from a local compilation of exposure-factor surveys by the Taiwan Department of Health (S4 Table)[46].

## Results and discussion

### Formaldehyde concentration reduction

**Fig 1:**
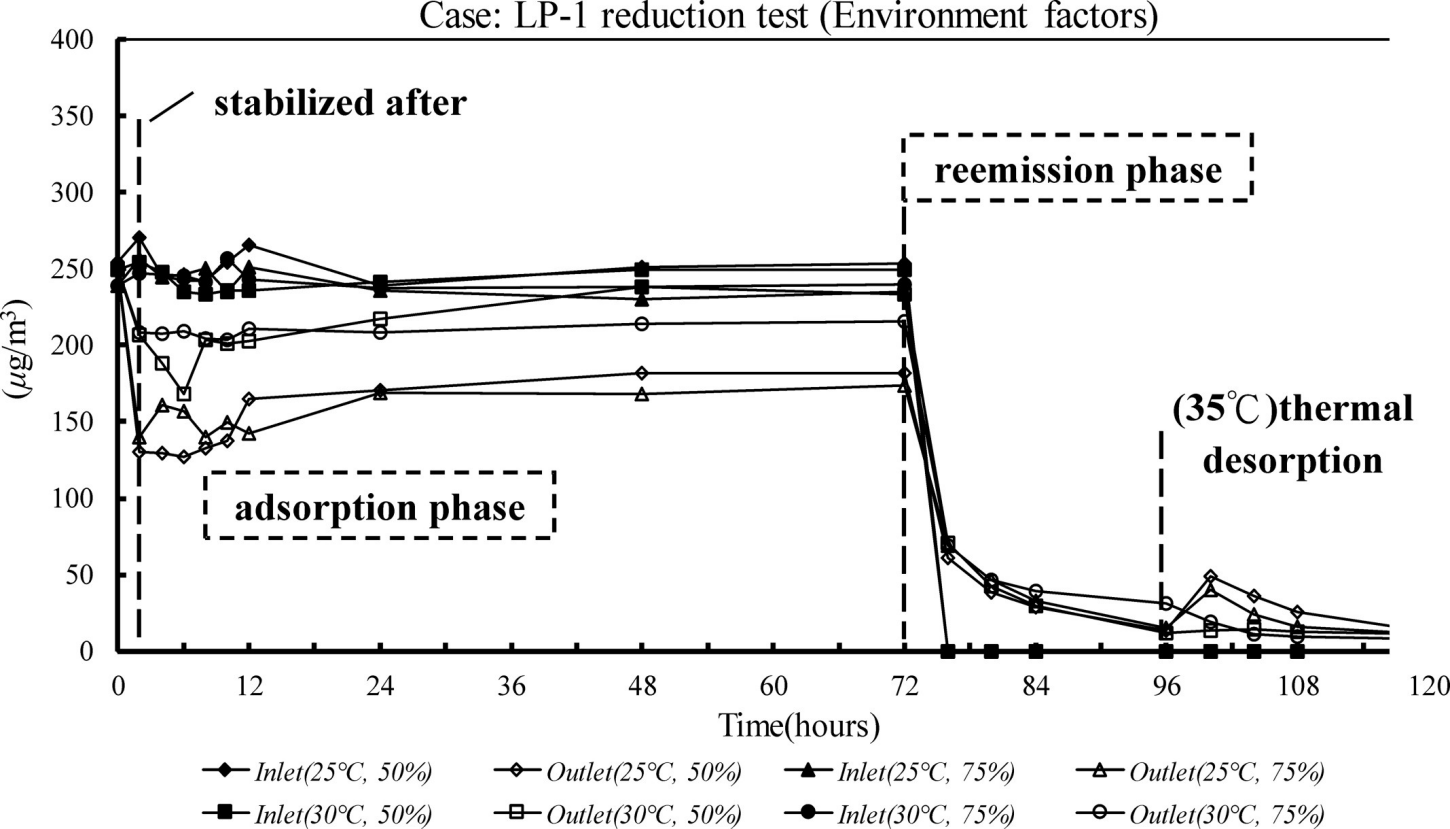
HCHO concentrations of the reduction test based on the LP-1 case environmental factors.

**Fig 2:**
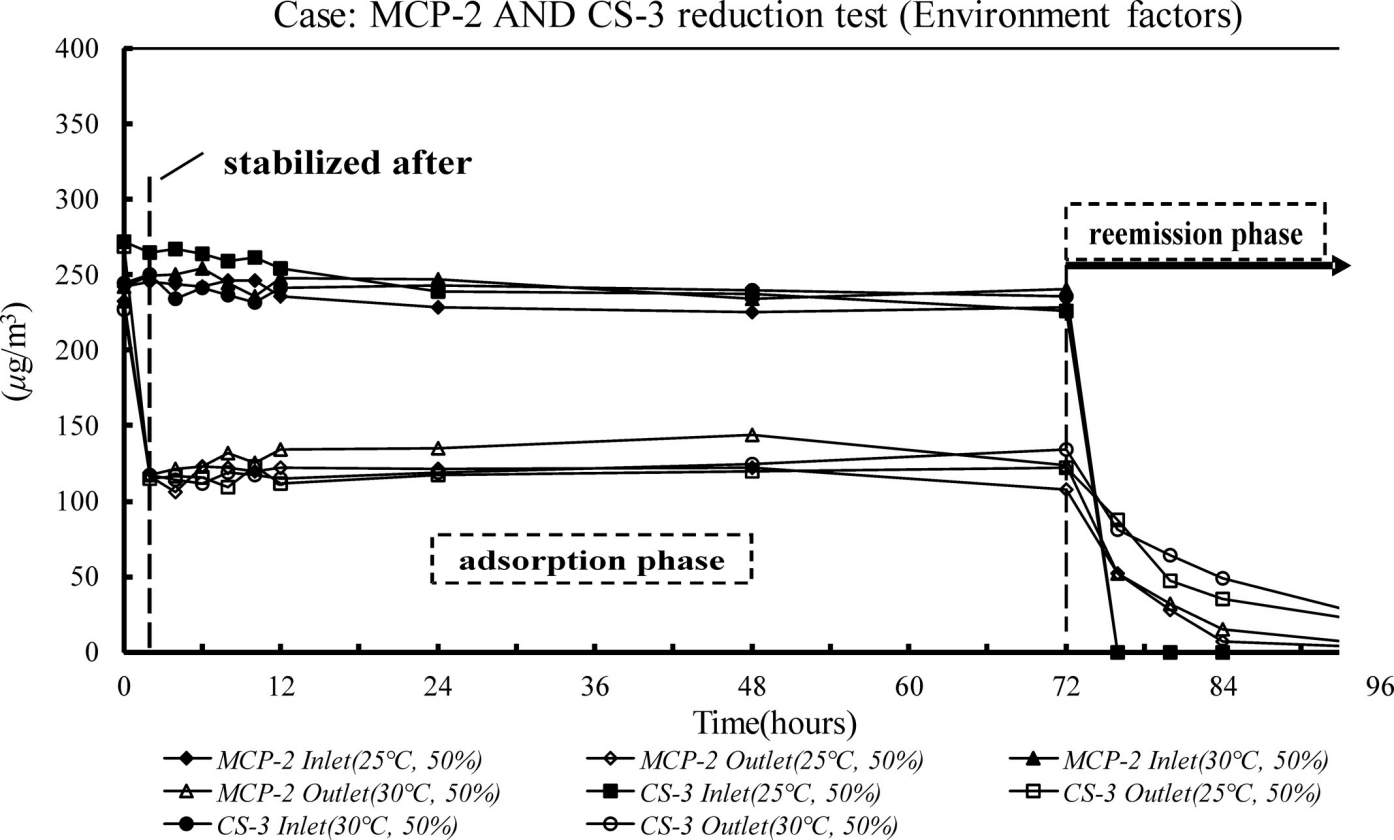
HCHO concentrations of the reduction tests based on the MCP-2 and CS-3 case environmental factors.

**Fig 3:**
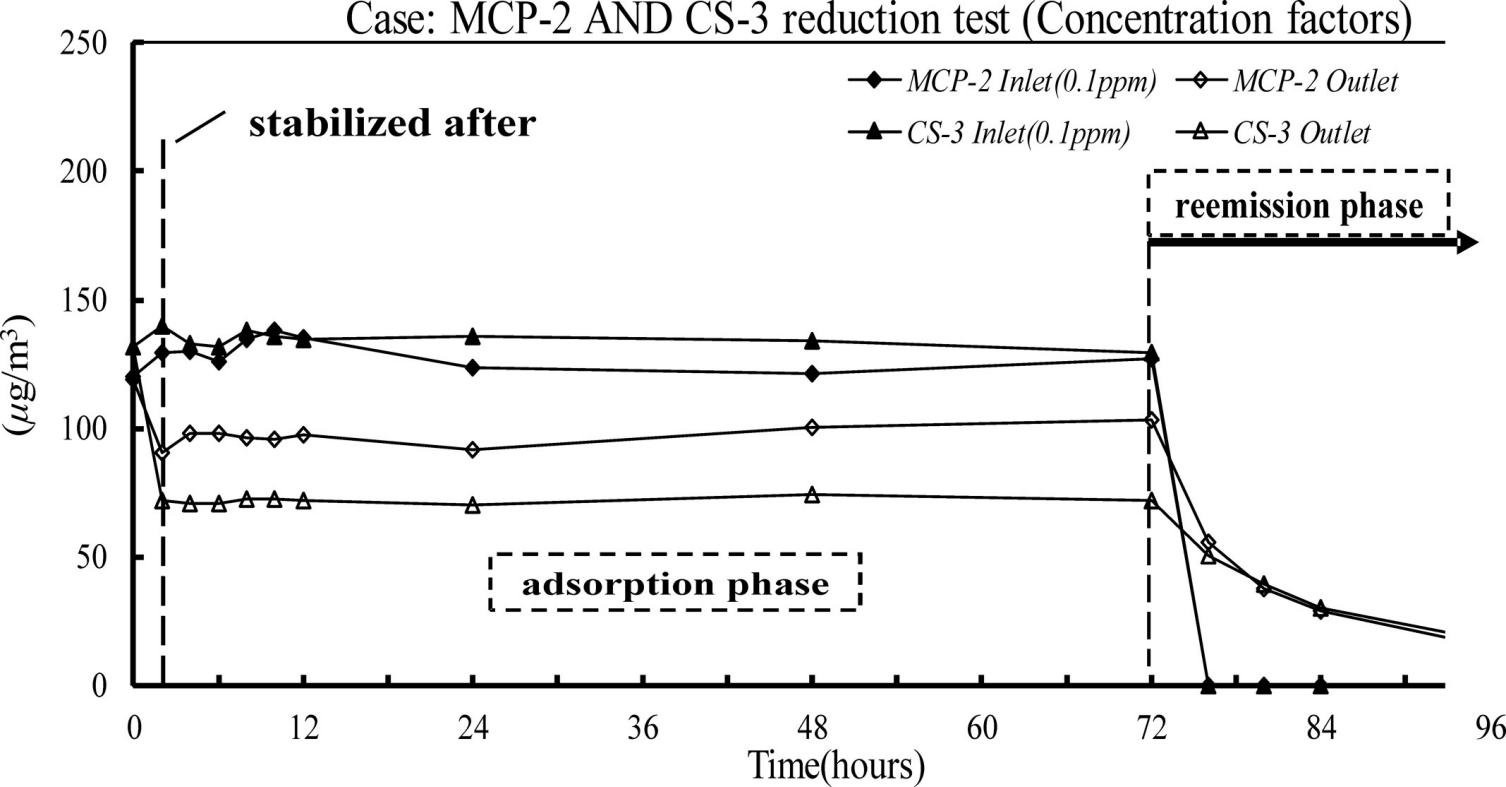
HCHO concentrations of the reduction tests based on a 0.1 ppm HCHO concentration for cases MCP-2 and CS-3.

There are several criteria for evaluating SBMs. First, the HCHO recovery in the inlet and outlet of the environmental chamber was > 80% before it contained test materials; this showed that there was no gas leak (Table 1). The SBM used in the experiment reduced HCHO concentrations in a specific environment, and it did not reemit absorbed HCHO in the reemission test. The adsorption in the LP-1 Case in high concentrations (0.2 ppm) changed with different environmental factors (Table 2 and Fig 1). When the temperature and humidity were high, the paint adsorbed significantly less HCHO; the adsorption rate was < 15%. The best environmental conditions for LP-1 were at 25°C and 50% RH. The average absorption rate was 40.1%, *SB*_m_ was 124.9 μg/m^2^·h, and *SB*_*v*,*eq*_ was 0.83 m^3^/m^2^·h, which was equal to the actual ACH rate of 0.33 with a loading factor of 0.4 m^2^/m^3^. There was no detectable HCHO release in any desorption stage (Fig 1). The effects of different temperatures and concentrations of airborne HCHO on the adsorption rates of MCP and CS boards were evaluated in the MCP-2 and CS-3 cases (Table 2 and Fig 2). There was no significant difference in the adsorption phase, but there was no detectable effect on the adsorptive ability of the boards at high temperatures. For both cases, the best adsorption rates were when the HCHO concentration was 0.2 ppm. The adsorption rates were 50.5% for the MCP-2 case and 53.8% for the CS-3 case. The *SB*_*v*,*eq*_ values, 1.26 and 1.44 m^3^/(m^2^·h), were equivalent to the effective ventilation rates of 0.50 h^‒1^ and 0.57 h^‒1^, respectively. At the low HCHO concentration (0.1 ppm), the adsorption rate in the MCP-2 case fell to 25.14%. In addition, the *SB*_*v*,*eq*,_ 0.42 m^3^/(m^2^·h), was not significantly different from the effective ventilation rate of 0.2 h^‒1^. There was no significant release of HCHO at any desorption stage (Figs 2 and 3). After 72 h had elapsed, the supply of HCHO gas was terminated and clean air was supplied at the inlet for the reemission test that followed.

**Table 1:** Sampling results of formaldehyde recovery over 72 h.

Test Conc.	Position	Time	Sampling	Measured	Recovery
			Vol. (m^3^)	Conc. (μg/m^3^)	
Formaldehyde	inlet	24 h	0.012	245	96%
0.2 ppm	outlet			234	
(244 μg/m^3^)	inlet	48 h		244	97%
	outlet			236	
	inlet	72 h		242	98%
	outlet			237	

Conc., concentration; Vol., volume.

**Table 2:** Sorption effectiveness of building materials.

Case	LP-1				MCP-2			CS-3		
Inlet concentration (ppm)	0.2	0.2	0.2	0.2	0.1	0.2	0.2	0.1	0.2	0.2
Temperature (°C)	25	25	30	30	25	25	30	25	25	30
RH (%)	50	75	50	75	50	50	50	50	50	50
*C*_*in*,*te*_ (μg/m^3^) ^a^	252(10.8)	243(9.3)	243(8.0)	244(5.9)	130(5.6)	238(8.6)	245(6.8)	135(3.1)	253(14.8)	239(5.6)
*C*_*out*,*te*_ (μg/m^3^)^a^	151(23.7)	156(13.2)	207(21.6)	209(4.0)	97(4.1)	118(6.5)	129(8.3)	72(1.3)	117(4.4)	119(6.8)
*SB*_m_ (μg/m^2^·h)	124.9	107.8	44.6	42.9	40.3	148.6	144.1	77.9	168.0	148.9
*SB*_*v*, *eq*_ (m^3^/m^2^·h)	0.83	0.69	0.22	0.21	0.42	1.26	1.12	1.08	1.44	1.25
Adsorption rate (%)	40.1	35.9	14.9	14.2	25.1	50.5	47.5	46.7	53.8	50.2
ER (mg/m^2^·h)	0.017	0.018	0.015	0.04	0.018	0.004	0.006	0.021	0.024	0.027

^a^Mean (standard deviation)

LP, latex paint; MCP, micro-carbonized plywood; CS, Celite siding; RH, relative humidity; *C*, formaldehyde concentration; _*in*_, inlet; _*out*_, outlet; _*v*_, volume; _*eq*_, equivalent; _*te*_, the time from the start of the test to the start of air sampling; *SB*_*m*_, sorption flux of building materials; ER, emission rate.

After the chamber air had been flushed and the SBMs had been adsorbing HCHO for 72–96 h, the chamber’s HCHO concentration gradually decreased, except in the MCP-2 case, in which it immediately decreased when the concentration was at 0.2 ppm. The emission rate (ER) of these three building materials were less than 0.05 mg/m^2^·h at 96 h, which fulfills the criteria for the Green Building Materials Label (GBML).

HCHO emission levels can be estimated using an environmental chamber, which is widely considered sufficiently accurate for evaluating the effect of HCHO emissions on human health [34]. Moreover, there are several standard quality control tests for wood-based materials EN 717–1, ASTM D 6007–02, and ASTM E 1333, which are used in developed countries [33–35].

To understand which factor controls HCHO emissions from dry building materials, three major emission parameters are usually discussed. They are the initial emittable concentration (*C*_*in*_), diffusion coefficient (*D*_*m*_), and partition coefficient (*K*) [47–50]. The correlation between *C*_*in*_ and the simultaneous effects of temperature and humidity are useful for simulating HCHO emissions in real buildings as well as developing green (low-emission) building materials. Matthews et al. (1987) used a single exponential model to evaluate the sorption and desorption processes of HCHO on gypsum boards, which significantly affected airborne HCHO concentrations because of its remarkable storage capacity [51].

Moreover, experimental studies have also showed an increase in HCHO emission rates as the temperature rose [52–57]. Zhang et al. (2007) reported a correlation between *K* and temperature [14], and two other studies reported correlations between *D*_*m*_ and temperature and between *C*_*in*_ and temperature for HCHO emissions [10, 58].

Some experimental studies also pointed that the emission rate and chamber concentration increased with increasing relative humidity (RH). Andersen et al. (1975) found that the emission rate of HCHO from particleboard doubled when the RH rose from 30% to 70% [52]. When it rose from 50% to 80%, the emission rates and chamber concentrations of certain VOCs (toluene: 3.5–5.4; n-butyl acetate: 1.1–1.4, ethylbenzene: 1.8–3.8, and m,p-xylene: 1.5–3.5 times) increased [54]. The HCHO emissions from a urea-formaldehyde (UF)-bonded particleboard from RH 30% to RH 75% doubled at 25°C and sextupled at 40°C [59]. Myers (1985) proposed an exponential relation between the steady-state HCHO emission rate and RH [53]. Prior studies reported that the HCHO emission rate had increased 6–9 times when RH increased from 30% to 100% [60, 61]. Parthasarathy et al. (2011) said that the steady-state HCHO emission rate increased by 1.8 to 3.5 times after a rise in RH from 50% to 85% [55].

Xiong & Zhang (2010) claimed that *C*_*in*_ increased by 507% when temperature increased from 25.2°C to 50.6°C [12]. These experimental studies have verified the positive effect of temperature on HCHO emissions. Based on the chamber measurement results with identical RH, a correlation between *C*_*in*_ and temperature was established [58].

Although many prior studies have reported the individual effects of temperature and humidity on HCHO emissions, the researchers were uncertain about how to use that information. Indoor temperature and humidity are generally not constant [62, 63] because they change with dweller behavior [64, 65]. The simultaneous effects rather than the individual effects of temperature and humidity need to be carefully evaluated when materials emit HCHO under different environmental conditions.

Because the CS-3 case continued for more than one week, we used the breakthrough test to verify the sustainability of the long-term adsorptivity of CS. We estimated it to be about 0.9 years (10.9 months). Because the other SBMs were not as sustainable, we did not use them for long-term tests.

### Health risk reduction

In typical dwellings in Taiwan with a floor area of 14 m^2^, a wall area of 45 m^2^, a volume of 42 m^3^, and a ventilation rate of 0.5 h^−1^[42], HCHO concentrations can be as high as 53.3 μg/m^3^, as in the LP-1 case (*C*_*in*,*te*_: 243.9 μg/m^3^, 30°C, 75% RH, ER: 0.04 mg/m^2^·h) (Table 3). The cancer risks ranged from 10.9 × 10^−6^ to 1.29 × 10^−4^, and most risks were greater than the acceptable risk 1 × 10^−6^. Except for the MCP-2 case (*C*_*in*,*te*_: 238 μg/m^3^, 25°C, 50% RH), the indoor HCHO concentrations of all buildings exceeded the acceptable inhalation risk level corresponding to a cancer risk of 100 excess cases per million (8 μg/m^3^) [27]. In addition, there was substantial risk reduction in the MCP-2 case (reduced HCHO concentration: *C*_*in*,*te*_ ‒ *C*_*steady*_ = 245 ‒ 8 = 237 μg/m^3^ at 30°C and 50% RH), which showed reductions of up to 5.73 × 10^−4^ in males and of up to 4.84 × 10^−4^ in females (Eq [7]). They represent a very high sorption performance and large reductions in the risk of formaldehyde inhalation by humans. The SBM, therefore, has a critical effect on the consequences of human inhalation of HCHO.

**Table 3:** Calculated mean formaldehyde human cancer risks in the presence of sorptive building materials.

Case	LP-1				MCP-2			CS-3		
Conc. (ppm)	0.2	0.2	0.2	0.2	0.1	0.2	0.2	0.1	0.2	0.2
Temp. (°C)	25	25	30	30	25	25	30	25	25	30
RH (%)	50	75	50	75	50	50	50	50	50	50
*C*_*in*,*te*_ (μg/m^3^)	251.6	242.7	242.5	243.9	129.5	238.0	245.0	134.8	252.6	239.4
ER (mg/m^2^·h)	0.017	0.018	0.015	0.04	0.018	0.004	0.006	0.021	0.024	0.027
*C*_*steady*_ (μg/m^3^)	22.7	24.0	20.0	53.3	24.0	5.3	8.0	28.0	32.0	36.0
Risk_*male*_ (×10^−6^)	54.8	58.0	48.3	128.9	58.0	12.9	19.3	67.7	77.3	87.0
Risk_*female*_ (×10^−6^)	46.3	49.0	40.8	108.9	49.0	10.9	16.3	57.2	65.4	73.5
Risk_*reduction*,*male*_ (×10^−6^)	553	528	538	460	255	562	573	258	533	491
Risk_*reduction*,*female*_ (×10^−6^)	468	447	454	389	215	475	484	218	451	415

Conc., concentration; Temp., temperature; RH, relative humidity; *C*, formaldehyde concentration; _in_, inlet; _*te*_, the time from the start of the test to the start of air sampling; ER, emission rate; LP, latex paint; MCP, micro-carbonized plywood; CS, Celite siding.

Our risk assessment procedure has some limitations. First, we used the average values of each parameter to calculate the single or fixed Inhalation Risk. In fact, Monte Carlo simulation, or probability simulation, has been recommended for understanding the distribution of risk.

Most people are concerned that the sorption capacity of building materials will decrease with time and eventually reach saturation. After that, the testing materials can inefficiently reduce indoor HCHO concentrations, but they will cause formaldehyde exposure due to the HCHO reemission from the materials. We found that the effective usage period of some SBMs was about 333 days (about 0.9 years), which is acceptable, because pollution sources are not normally permanently continuous emissions, and because the real-life mission concentrations dwellings and office buildings are usually much lower than those in the testing laboratories and some production facilities. The emission source in typical indoor environment is periodic and lower, e.g., the micro-dissipation of related electronic products and some furniture. However, when there is long-term absorption and desorption of VOCs, building occupants should open the windows to increase the ventilation rate and reduce or eliminate accumulated VOCs.

## Conclusions

We conclude that the SBM test system (SBMT) is efficacious and that our findings confirm that using green building materials for interior decoration materials reduces the accumulation of harmful pollutants. High temperature and high humidity significantly reduced the adsorption ability of LP.

MCP rapidly adsorbed a great deal of HCHO and significantly reduced the risk of human inhalation of HCHO indoors. In typical Taiwanese dwellings, CS was an effective SBM for up to 333 consecutive days. The selection of SBM, therefore, is important. Future studies are warranted to verify the efficacity of these and other SBMs.

## Supporting information

S1 Fig Evaluating the sorption/desorption performance test in the concept. (TIF)

S2 Fig Sorptive building material test system (SBMTS). (TIF)

S3 Fig Long-term monitoring of formaldehyde recovery. (TIF)

S4 Fig Long-term reduction test of HCHO concentrations at 0.1 ppm for case CS-3. (TIF)

S1 Table Standard test method of the sorption of building materials. (DOCX)

S2 Table Conditions for air sampling test in a small-scale chamber. (DOCX)

S3 Table Measurement samples and conditions. (DOCX)

S4 Table Risk parameters of this study. (DOCX)

S5 Table Long-term sorption effectiveness of building materials. (DOCX)

S6 Table The long-term effective duration of building materials. (DOCX)

S1 File The raw data of HCHO risk. (XLSX)

## Abbreviations

CS: Celite siding
GBML: green building materials label
HCHO: formaldehyde
L: loading factor
LP: latex paint
MBS: moisture-buffering siding
MCP: micro-carbonized plywood
RH: relative humidity
IAQ: indoor air quality
IEH: indoor environmental health
SBMT: sorptive building materials test
VOCs: volatile organic compounds
CNS: Chinese National Standards
