# Supplementary figures and images for: Efficiency and performance tests of the sorptive building materials that reduce indoor formaldehyde concentrations
Source: PLoS One. 2019 Jan 24;14(1):e0210416. doi: 10.1371/journal.pone.0210416 (PMC6345484; doi:10.1371/journal.pone.0210416)

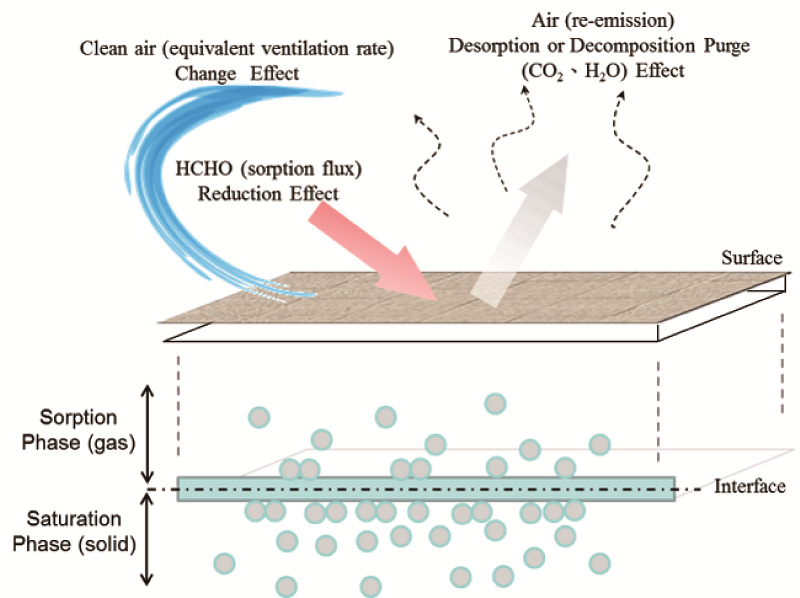

Supplement: S1 Fig — (TIF) [file pone.0210416.s001.tif]

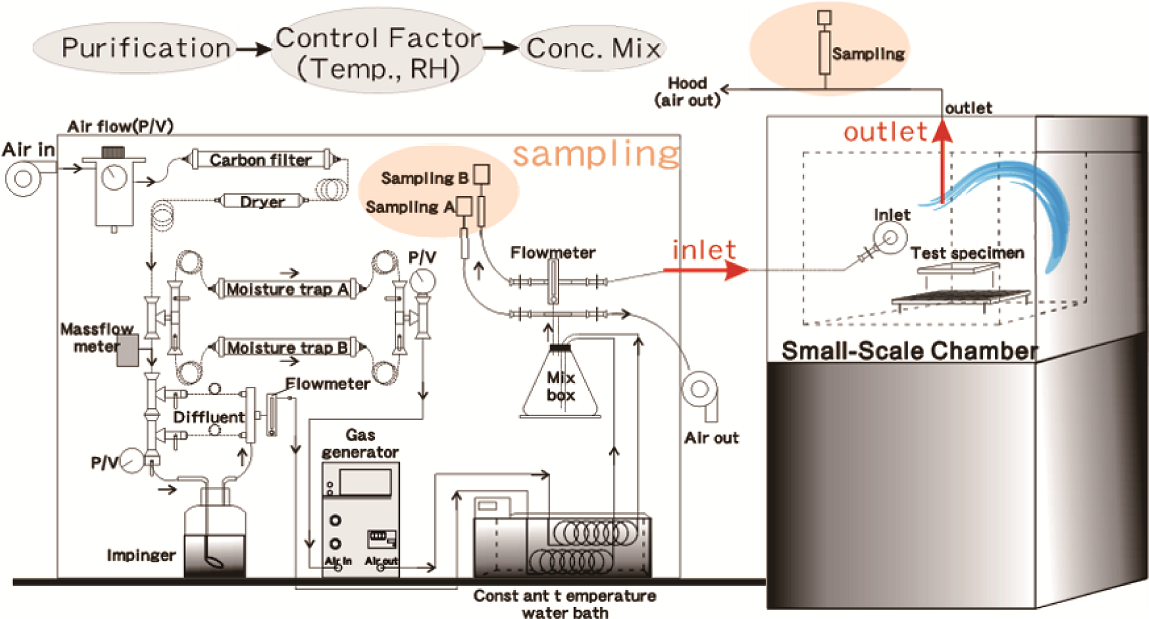

Supplement: S2 Fig — (TIF) [file pone.0210416.s002.tif]

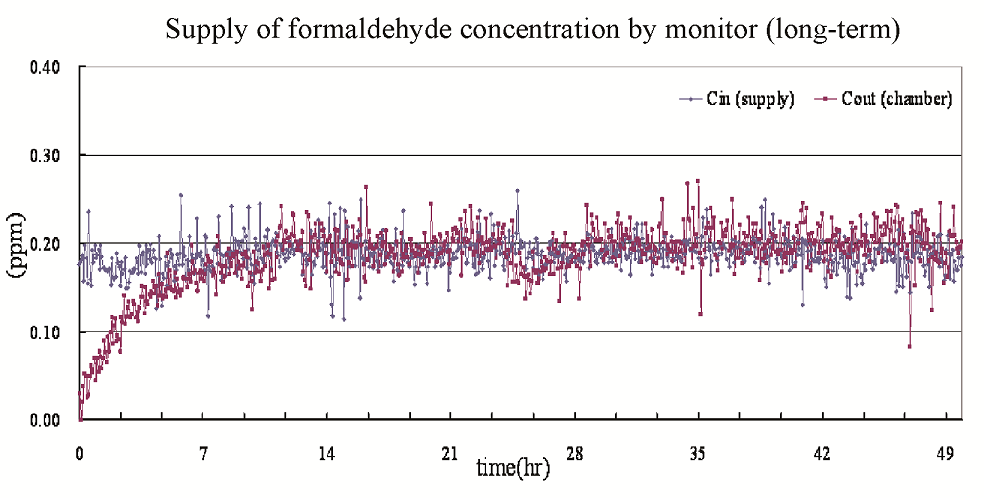

Supplement: S3 Fig — (TIF) [file pone.0210416.s003.tif]

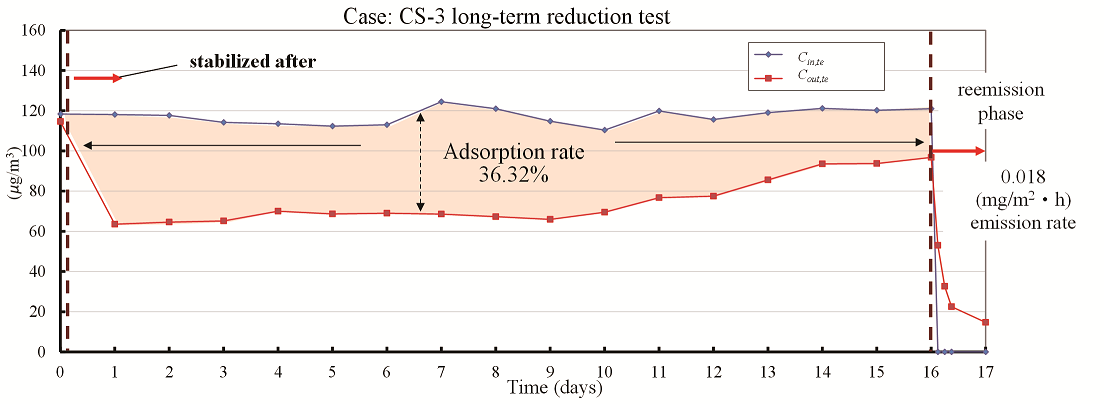

Supplement: S4 Fig — (TIF) [file pone.0210416.s004.tif]
