# Supplementary material for: Efficiency and performance tests of the sorptive building materials that reduce indoor formaldehyde concentrations
Source: PLoS One. 2019 Jan 24;14(1):e0210416. doi: 10.1371/journal.pone.0210416 (PMC6345484; doi:10.1371/journal.pone.0210416)
Supplement: S1 Table — (DOCX) [file pone.0210416.s005.docx]

**S1 Table. Standard test method of the sorption of building materials**

| **Reduction indoor VOC concentration standards** | | | |
| --- | --- | --- | --- |
| Type | ISO | JIS (Japan) | CNS (Taiwan) |
| *HCHO* | ISO 16000-23 (2009) | JIS A 1905-1 (2007) | CNS 16000-23 (2011) |
| *VOCs* | ISO 16000-24 (2009) | JIS A 1906 (2008) | CNS 16000-24 (2011) |
| *Other* | ─ | JIS A 1905-2 (2007) | ─ |
| *Appraise* | ─ | BCJ-CS-5 (2003) | ─ |

HCHO, formaldehyde. VOCs, Volatile Organic Compounds
