# Supplementary material for: Efficiency and performance tests of the sorptive building materials that reduce indoor formaldehyde concentrations
Source: PLoS One. 2019 Jan 24;14(1):e0210416. doi: 10.1371/journal.pone.0210416 (PMC6345484; doi:10.1371/journal.pone.0210416)
Supplement: S2 Table — (DOCX) [file pone.0210416.s006.docx]

**S2 Table. Conditions for air sampling test in a small-scale chamber**

| **Sampling** | **Small-scale Chamber (*Volume*: 99 L)** |
| --- | --- |
| *Tube:* DNPH (derivatives) | **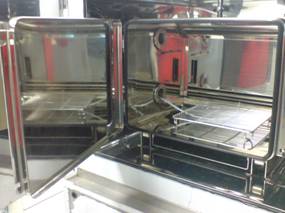** |
| *Flow:* 100 mL/min |  |
| *Volume:* 12 L (2 h) |  |
| *Menstruum:* Acetonitrile |  |
| *Analysis:* HPLC |  |
| *Quantitative:* Calibration curve |  |

DNPH, 2,4-dinitrophenyl hydrazine; HPLC, high performance liquid chromatography.
