# Supplementary material for: Efficiency and performance tests of the sorptive building materials that reduce indoor formaldehyde concentrations
Source: PLoS One. 2019 Jan 24;14(1):e0210416. doi: 10.1371/journal.pone.0210416 (PMC6345484; doi:10.1371/journal.pone.0210416)
Supplement: S3 Table — (DOCX) [file pone.0210416.s007.docx]

**S3 Table. Measurement samples and conditions**

| **Performance test of sorptive building materials** | |
| --- | --- |
| **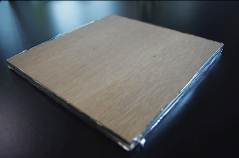**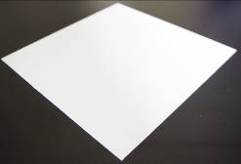GBMs (Taiwan) | HCM (Japan) |
|  | 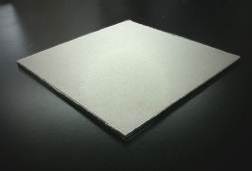 |
| LP-1 MCP-2 | CS-3 |
| **Products Case** | |
| *A*: 0.2 m × 0.2 m (*L*: 0.4m^2^/m^3^) | Health of Low emission |
| ***Environmental effect*** | |
| 25°C, 50% RH (Guideline) | 30°C, 75% RH (Suggested) |
| ***Formaldehyde concentration*** | |
| 0.1 ppm: 122μg/m^3^ (WHO Guideline) | 0.2 ppm: 244 μg/m^3^ (Twice) |
| **Experimental conditions in a small-scale chamber** | |
| *Air change rate:* 0.5/h ± 0.05/h |  |
| *Background:* VOC < 2 μg/m^3^ | TVOC < 20 μg/m^3^ |
| *Mass transfer:* 0.25 m/s ± 0.05 m/s (15 m/h ± 3 m/h) |  |
