# Supplementary material for: Efficiency and performance tests of the sorptive building materials that reduce indoor formaldehyde concentrations
Source: PLoS One. 2019 Jan 24;14(1):e0210416. doi: 10.1371/journal.pone.0210416 (PMC6345484; doi:10.1371/journal.pone.0210416)
Supplement: S4 Table — (DOCX) [file pone.0210416.s008.docx]

**S4 Table. Risk parameters of this study**

| Input parameters | Adult Males | Adult Females |
| --- | --- | --- |
| Average Life Span (years) | 76.2 | 83.0 |
| the time of exposure (hour/day) | 14 | 14 |
| the duration of exposure (years) | 70 | 70 |
| the average lifetime (years) | 77 | 84 |

Data are from Department of Health (DOH), Taiwan. 2008.

**S4 Table. Risk parameters of this study (cont’d)**

|  |  |  | Inhalation Rates (m^3^/day) | | |
| --- | --- | --- | --- | --- | --- |
|  | AGE (yr) | BW (kg) | Inactive | | Active |
| male | 0 | 9.08 | 5.9^a^ | | 13.97^a^ |
|  | 1 | 11.84 | 5.9^a^ | | 13.97^a^ |
|  | 2 | 14.38 | 5.9^a^ | | 13.97^a^ |
|  | 3 | 16.11 | 5.9^a^ | | 13.97^a^ |
|  | 4 | 18.43 | 5.9^a^ | | 13.97^a^ |
|  | 5 | 20.75 | 5.9^a^ | | 13.97^a^ |
|  | 6 | 23.79 | 5.9 | | 13.97 |
|  | 7 | 26.92 | 6.3 | | 14.6 |
|  | 8 | 31.38 | 6.87 | | 13.33 |
|  | 9 | 34.37 | 7.26 | | 18.79 |
|  | 10 | 38.64 | 7.8 | | 19.26 |
|  | 11 | 42.12 | 7.93 | | 19.41 |
|  | 12-14 | 53.2 | 9.03 | | 18.31 |
|  | 15-19 | 63.94 | 9.35 | | 19.68 |
|  | 20-24 | 68.57 | 9.74 | | 18.42 |
|  | 25-34 | 71.38 | 9.56 | | 20.01 |
|  | 35-54 | 71.72 | 9.58 | | 16.57 |
|  | 55-64 | 67.19 | 9.29 | | 16.81 |
|  | 65-69 | 61.9 | 9.29^b^ | | 16.81^b^ |
|  | 70-74 | 61.25 | 9.29^b^ | | 16.81^b^ |
|  | 75-76 | 60.57 | 9.29^b^ | | 16.81^b^ |
|  | | | |  |  |
|  | | | |  |  |

a: the data is not available and assumed to be the same as 6 yrs

b: the data is not available and assumed to be the same as 55-64 yrs

**S4 Table. Risk parameters of this study (cont’d)**

|  |  |  | Inhalation Rates (m^3^/day) | |
| --- | --- | --- | --- | --- |
|  | AGE (yr) | BW (kg) | Inactive | Active |
| female | 0 | 8.21 | 5.29^c^ | 14.02^c^ |
|  | 1 | 10.79 | 5.29^c^ | 14.02^c^ |
|  | 2 | 13.91 | 5.29^c^ | 14.02^c^ |
|  | 3 | 16.3 | 5.29^c^ | 14.02^c^ |
|  | 4 | 17.57 | 5.29^c^ | 14.02^c^ |
|  | 5 | 19.7 | 5.29^c^ | 14.02^c^ |
|  | 6 | 22.15 | 5.29 | 14.02 |
|  | 7 | 25.78 | 5.7 | 11.7 |
|  | 8 | 29.04 | 6.08 | 13.37 |
|  | 9 | 33.1 | 6.54 | 16.34 |
|  | 10 | 37.18 | 7.01 | 16.81 |
|  | 11 | 40.13 | 6.95 | 18.05 |
|  | 12-14 | 46.86 | 7.45 | 12.48 |
|  | 15-19 | 52.47 | 7.14 | 12.62 |
|  | 20-24 | 52.46 | 7.14 | 11.86 |
|  | 25-34 | 58.61 | 7.47 | 11.79 |
|  | 35-54 | 59.38 | 7.5 | 13.72 |
|  | 55-64 | 61.07 | 7.58 | 11.54 |
|  | 65-69 | 65.99 | 7.58^d^ | 11.54^d^ |
|  | 70-74 | 64.06 | 7.58^d^ | 11.54^d^ |
|  | 75-79 | 64.8 | 7.58^d^ | 11.54^d^ |
|  | 79-83 | 62.26 | 7.58^d^ | 11.54^d^ |

c: the data is not available and assumed to be the same as 6 yrs

d: the data is not available and assumed to be the same as 55-64 yrs
