# Supplementary material for: Efficiency and performance tests of the sorptive building materials that reduce indoor formaldehyde concentrations
Source: PLoS One. 2019 Jan 24;14(1):e0210416. doi: 10.1371/journal.pone.0210416 (PMC6345484; doi:10.1371/journal.pone.0210416)
Supplement: S5 Table — (DOCX) [file pone.0210416.s009.docx]

**S5 Table. Long-term sorption effectiveness of building materials.**

| **Case** |  | **CS-3** | |
| --- | --- | --- | --- |
| Concentration (ppm) |  | 0.1 |  |
| Temperature (°C) |  | 25 |  |
| RH (%) |  | 50 |  |
| *C_in,te_* (μg/m^3^) |  | 117.28 |  |
| *C_out,te_* (μg/m^3^) |  | 74.76 |  |
| *SB*_m_ (μg/m^2^·h) |  | 52.61 |  |
| *SB_v_*_,_ *_eq_* (m^3^/m^2^·h) |  | 0.74 |  |
| Adsorption rate (%) |  | 36.32 |  |
| ER (mg/m^2^·h) |  | 0.018 |  |

CS, Celite™ siding ; RH, relative humidity ;*C*, formaldehyde concentration ; *_te_*, the time from the start of the test to the start of air sampling; *SB*, sorption flux of building materials; ER, emission rate.
