# Supplementary material for: Efficiency and performance tests of the sorptive building materials that reduce indoor formaldehyde concentrations
Source: PLoS One. 2019 Jan 24;14(1):e0210416. doi: 10.1371/journal.pone.0210416 (PMC6345484; doi:10.1371/journal.pone.0210416)
Supplement: S6 Table — (DOCX) [file pone.0210416.s010.docx]

**S6 Table. The long-term effective duration of building materials.**

| 25℃, formaldehyde 122 μg/m^3^(0.1 ppm) | | | | | |
| --- | --- | --- | --- | --- | --- |
| Elapsed time, Δ*t*_e,i_ (days) | Chamber  ρ*_Ac_*(μg/m^2^) | Chamber  *SB_m_*(μg/m^2^/h) | *Sample tube Test W_s_*(μg/g) | *Sample tube Test ρ_Aa_*(μg/m^2^) | *t_lt_*  (day) |
| CS-3  (16 days) | 20202.19 | 52.61 | 74.84 | 420975 | 333(0.9) |
